# The Wound Healing Potential of* Aspilia africana* (Pers.) C. D. Adams (Asteraceae)

**DOI:** 10.1155/2019/7957860

**Published:** 2019-01-21

**Authors:** Richard Komakech, Motlalepula Gilbert Matsabisa, Youngmin Kang

**Affiliations:** ^1^University of Science & Technology (UST), Korea Institute of Oriental Medicine (KIOM) Campus, Korean Medicine Life Science Major, Daejeon 34054, Republic of Korea; ^2^Herbal Medicine Resources Research Center, Korea Institute of Oriental Medicine, 111 Geonjae-ro, Naju-si, Jeollanam-do 58245, Republic of Korea; ^3^Natural Chemotherapeutics Research Institute (NCRI), Ministry of Health, P.O. Box 4864, Kampala, Uganda; ^4^University of the Free State, 205 Nelson Mandela Drive, Bloemfontein 9300, South Africa

## Abstract

Wounds remain one of the major causes of death worldwide. Over the years medicinal plants and natural compounds have played an integral role in wound treatment.* Aspilia africana* (Pers.) C. D. Adams which is classified among substances with low toxicity has been used for generations in African traditional medicine to treat wounds, including stopping bleeding even from severed arteries. This review examined the potential of the extracts and phytochemicals from* A. africana*, a common herbaceous flowering plant which is native to Africa in wound healing.* In vitro* and* in vivo* studies have provided strong pharmacological evidences for wound healing effects of* A. africana*-derived extracts and phytochemicals. Singly or in synergy, the different bioactive phytochemicals including alkaloids, saponins, tannins, flavonoids, phenols, terpenoids, *β*-caryophyllene, germacrene D, *α*-pinene, carene, phytol, and linolenic acid in* A. africana* have been observed to exhibit a very strong anti-inflammatory, antimicrobial, and antioxidant activities which are important processes in wound healing. Indeed,* A. africana* wound healing ability is furthermore due to the fact that it can effectively reduce wound bleeding, hasten wound contraction, increase the concentration of basic fibroblast growth factor (BFGF) and platelet derived growth factor, and stimulate the haematological parameters, including white and red blood cells, all of which are vital components for the wound healing process. Therefore, these facts may justify why* A. africana* is used to treat wounds in ethnomedicine.

## 1. Introduction

A wound can be defined as the disruption of living tissue integrity associated with loss of function [1]. The wound healing process is a complex dynamic process which represents an attempt to restore a normal anatomical structure and function [2, 3]. Wounds can be broadly categorized as acute wounds which are caused by external injury to the skin and include surgical wounds, bites, burns, minor cuts and abrasions, and more severe traumatic wounds such as lacerations and those caused by crush or gunshot injuries or chronic etiology wounds which includes vascular, diabetic, and pressure ulcers [1, 4]. In fact, wounds impose significant health, social, and economic burdens to the individuals, the healthcare system, and the community as a whole [5, 6]. Recent statistics showed that approximately 3% of the healthcare budget is spent on treating wound-related complications in developed countries [6]. The aim of treating a wound is to prevent pain discomfort to the patient and promote wound healing which occurs mainly in four phases: hemostasis, inflammation, proliferation, and remodeling [1, 7, 8]. Plants have immense potential that can be explored for the treatment and management of wounds [2, 9]. Indeed, several medicinal plants have been used in traditional medicine for the treatment and management of all kinds of wounds across the globe since time immemorial [3, 10, 11].* Aspilia africana* (Pers.) C. D. Adams (Asteraceae), commonly referred to as wild sunflower, is one of the highly valued wound healing plants throughout its distribution range and beyond [12–14]. This unique wound healing plant species is commonly referred to as “haemorrhage plant” due to its distinguished ability to stop bleeding, even of severed artery [15, 16]. Apart from its enormous potential in wound healing,* A. africana* is reported to be vital in the treatment and management of myriad of other diseases and disorders in African traditional medicine, including headache, corneal opacities, stomach disorders, cough, gonorrhea, rheumatic pains, and tuberculosis; the leaf infusion is taken as a tonic for women immediately after delivery [17, 18].* A. africana* plant is known to possess great anti-inflammatory, antimalarial, and antimicrobial activities [12, 16]. Several scientific studies have attributed the numerous medicinal properties of* A. africana* to the abundant bioactive secondary metabolites in it such as alkaloids, saponins, tannins, glycosides, flavonoids, and terpenoids [18, 19].

The use of* A. africana* in wound treatment and management has been assessed and discussed in a number of peer reviewed journal articles over the years. This review therefore sought to examine the wound healing potential of* A. africana* both* in vitro* and* in vivo* with the goal of finding new drugs for treatment and management of wounds.

## 2. Methods

In this review, we obtained information from original peer reviewed articles published in scientific journals, with a focus on the botany, distribution, and potential of* A. africana *for treatment and management of wounds. We critically searched electronic literature databases including but not limited to Google Scholar, PubMed, and Scopus for all available peer reviewed data. The following key search terms were used (“*A. africana*” OR “Wild sunflower” AND “wounds” OR “wound healing” OR “Phytochemicals”) OR (“Phytochemicals in* A. africana*” OR “Wild sunflower” AND “wound” OR “wound healing”), OR (“Phytochemicals in* A. africana*” OR “Wild sunflower” AND “Anti-inflammation” OR “Anti-microbial”), OR (“Plants” OR “Natural products” AND “wound” OR “wound healing”) OR (“*A. africana*” OR “Wild sunflower” AND “Botany” OR “Distribution”). The data obtained were verified independently for their accuracy and any inconsistencies were settled through discussions between the authors. The final data obtained through discussions among the authors were then summarized, analyzed, and compared, and conclusions were made accordingly.

## 3. Botany and Distribution of* Aspilia africana*

**Figure 1:**
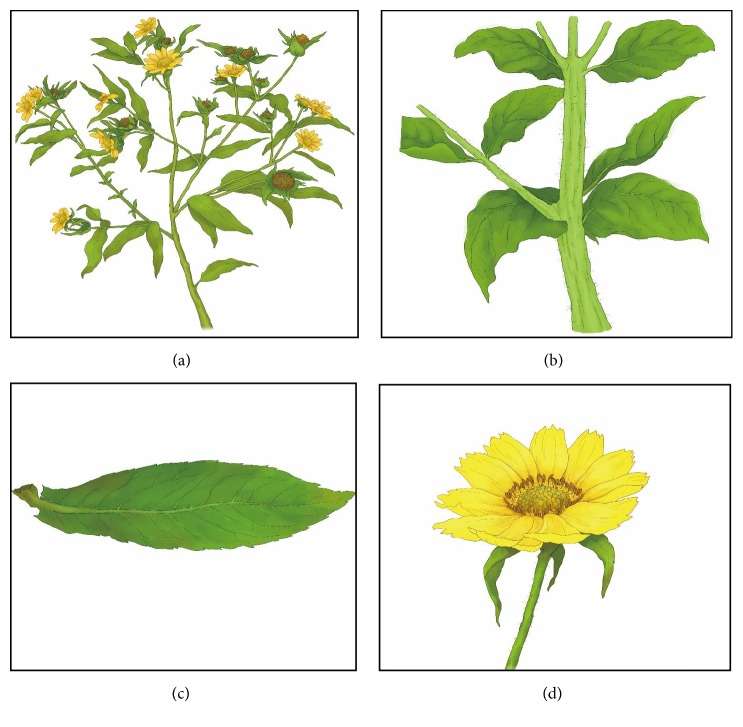
The morphological illustration of the main features of* A. africana*. (a)* A. africana* plant with numerous branches. (b)* A. africana* stem with numerous bristles. (c) Simple leaf of* A. africana, *oppositely arranged on the plant. (d) Inflorescence of* A. africana *consisting of outer ray and inner disc florets.

The genus* Aspilia *is a genus of common herbaceous flowering plants which are native to Africa and comprised of approximately 140 species [18, 64]. Morphologically,* A*.* africana* is a herb measuring about 1-2 m in height covered with bristles; stem is stiff at the base, with many branches and rough to touch (Figures 1(a) and 1(b)); leaves are rough, opposite, ovate-lanceolate, creased accordion-style covered with trichomes, average 10 cm long and 5 cm wide, and rounded at the base with petioles about 1 cm long with 3 prominent veins (Figure 1(c)); inflorescence consists of capitula which is terminal, solitary, or in lax racemes with hairy stalk of about 7 cm long on average; flowers have numerous showy-yellow florets; fruits are 4-angled achenes (Figure 1(d)) [12, 64, 65].

**Figure 2:**
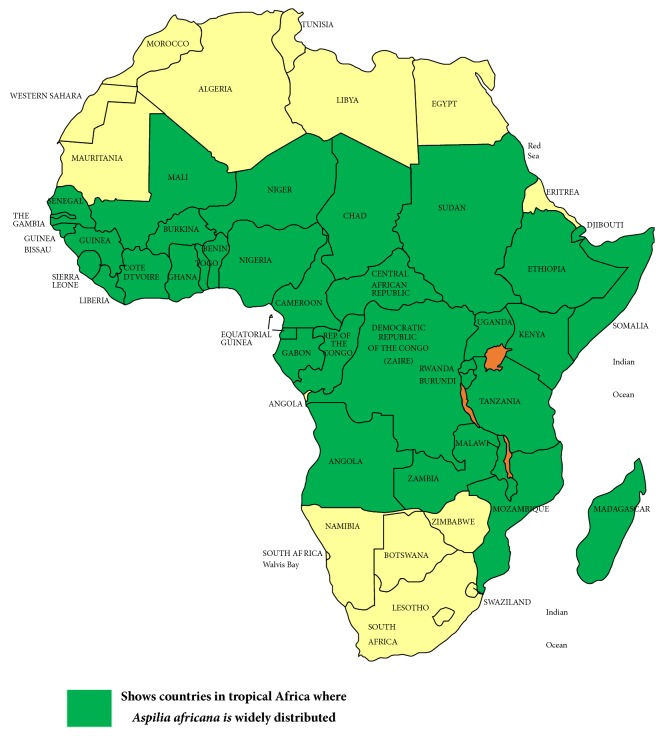
Modified map on distribution of* A. africana *[65].

*A. africana* is native to Africa occurring in a number of countries throughout the tropical African region on waste land of the savanna and forested zones between altitude of 800 and 1800 m (Figure 2), and its rapid growth characteristics make it a difficult weed in cultivated land and fallows [65].

## 4. Toxicological Effects of* Aspilia africana*

Generally, this unique wound healing plant can be classified among agents with low toxicity [66]. In an* in vivo* study by Okokon et al. [67] using Swiss albino mice, the acute toxicity of the ethanolic extract of the plant showed that doses of 2000 mgkg^−1^ and above were lethal to the animals and the determined LD_50_ of the extract was 1414.2 mgkg^−1^. Further,* in vivo* study by Oko et al. [68] on Swiss albino mice concluded that oral administration of up to 10,000 mgkg^−1^ body weight of aqueous and ethanolic extracts of the plant was safe for animal and human use. However, a recent study showed that the aqueous leaf extract of* A. africana* may be teratogenic to the developing placenta of Wistar rats in a dose-dependent manner; more severe outcomes were observed in female rats that received up to 1250 mg/kg body weight of the aqueous extract [69]. Similarly, other previous studies also showed that intraperitoneal administration of the extracts of* A. africana* leaf caused significant delay in estrus cycle and in addition did not only distort the histology of ovaries and reduce its weight, but also damaged the uterine tissues and fallopian tubes in Wistar rats [17, 67, 70, 71]. Furthermore, methanolic extracts of* A. africana* have also been found to significantly decrease the weight of testis, epididymis, seminal vesicle, and prostate gland of experimental male Wistar rats [72]. Therefore, despite the safety associated with* A. africana*, caution must be taken during its long term oral consumption as it may have adverse effects on reproductive organs.

## 5. Effects of Leaf Extracts of* Aspilia africana* on Wound Healing

*A. africana* is one of the many medicinal plants containing large quantities of bioactive compounds making it such a potent plant in wound sepsis treatment and management and other microbe induced disease conditions [19, 20]. Over the years, several* in vitro* and* in vivo* scientific studies have been conducted to validate the wound healing ability of this plant. In an* in vivo* study by Eweka and Eweka [73]; they examined the effects of aqueous extract of* A. africana* administered orally for fourteen days on the duodenum of adult Wistar rats exposed to varied concentrations of hydrochloric acid. The histological findings indicated sections of the small intestine of treated rats showed varying degrees of cellular proliferation and epithelia regeneration. This showed that* A. africana* consumption may have antiulcer effects on duodenal ulcer by its healing effects on the Brunnals gland and epithelia cells of the small intestine of adult Wistar rats. Similarly, earlier study by Nguelefack et al. [74] also showed that the methanolic extract of fresh leaves of* A. africana* at the dose of 1 g/kg reduced gastric lesion in the pylorus ligated rats by 52%, a further proof of the potential of* A. africana* in wound healing. In a study by Attama et al., 2011 [75] where they examined the methanol leaf extract of* A. africana* formulated as gels for its potency on experimentally induced wound in rats, 100% wound closure was observed by the 17th day of treatment in both gel formulations of the plant methanolic extract and the standard gel, an indication of effectiveness of* A. africana* in wound healing. Similarly, a study by Osunwoke et al. [76] on the wound healing effects of the leaves extract of* A. africana *on Wistar rats showed that the rate of contraction of the excised wounds in the experimental group on days 6 and 9 was significant (*P*<0.05) with a mean wound closure of 12.6±1.17 cm compared to those in the control group which was 15.0±1.86 cm. Furthermore, they observed that the concentration of neutrophils and macrophages was intense in the experimental group relative to than the control group in the excised tissue samples. The total wound closure and increased inflammatory response suggests that the aqueous extract of the leaves of* A. africana *promotes wound healing activity through increased inflammatory response and neovascularization. In another* in vivo* experimental evaluation by Okoli et al. [12] using Wistar rats, they observed that the methanolic and hexane extracts and methanolic fractions of* A. africana *significantly (*P*<0.05) reduced bleeding (clotting time) in the rats and caused varying degrees of inhibition of the growth of microbial organisms known to cause wound infections such as* Pseudomonas fluorescens *and* Staphylococcus aureus*. The study showed that the extracts reduced epithelialization period of wounds that were experimentally excised in the rats, hence validating the fact that* A. africana *possesses constituents capable of accelerating wound healing. At different concentrations,* A. africana* also showed varied stimulating effects on haematological parameters including white and red blood cells due to the enormous micronutrients found in the plant [77]. Indeed, increased haematological changes especially in the red blood cells count are known to result in increased level of oxygen supply to the wounds resulting in faster wound healing [78]. Additionally, the wound healing ability of* A. africana* has also heavily been attributed to its anti-inflammatory activity resulting in inhibition of prostaglandins synthesis, decreased vascular permeability, inhibition of neutrophil migration into inflamed tissues, and stimulation of lymphocyte accumulation, thus enhancing tissue repair and healing [12]. Indeed, anti-inflammatory activity is essential for wound healing, since a long duration of the inflammatory phase causes delay in the wound healing process [79]. Additionally, the strong antimicrobial activities of* A. africana* play a vital role in the ability of this plant to heal wound sepsis [80–84]. In fact, a study by Anibijuwon et al. [85] showed that* A. africana* has strong antimicrobial activities. These findings further showed that the anti-inflammatory and antimicrobial agent play vital roles in wound healing process.

## 6. The Potential of the Phytochemicals from* Aspilia africana *in Wound Healing

**Figure 3:**
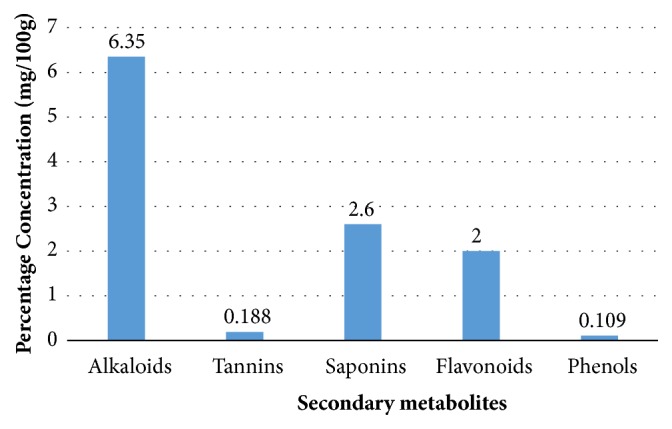
The percentage composition of phytochemical analysis of some of the nonvolatile secondary metabolites in the leaf extract of* A. africana* [88].

As discussed above,* in vivo* studies have provided strong pharmacological evidence for wound healing potential of the extracts obtained from* A. africana.* The plant is endowed with myriad of classes of bioactive secondary metabolites including alkaloids, saponins, tannins, flavonoids, and phenols (Figure 3) [12, 18, 20, 86, 87] and terpenoids [19, 20].* A. africana* also contains a number of other compounds (Table 1) such as sesquiterpenes including *β*-caryophyllene and germacrene D, and linolenic acid [20]. The presence of these phytochemicals suggests that* A. africana* might be of medicinal importance and supports the basis for its use in ethnomedicine as a wound healing plant.

The high content of alkaloids in* A. africana* may be one of the major contributing factors to the wound healing activity of this plant [64, 68]. A number of alkaloids have been known to have great wound healing activities [18]. In an* in vivo* study, topical application of an alkaloid enriched-ointment exhibited higher dermal healing activity of the wounds on rats [45]. Similarly, alkaloids have been observed to promote early phases of wound healing in a dose-dependent manner with the ability to stimulate chemotaxis for fibroblasts* in vitro* [89]. Alkaloids have also been observed to enhance significant wound healing activity (*P*<0.05) as evidenced by the increased rate of wound contraction and reduction in the period of epithelialization [90]. Sahib et al. [21] reported that the wound healing potential of* Ruta graveolens* L. plant may be due to the presence of alkaloids. These findings therefore suggest that the wound healing potential of* A. africana* may be due to the presence of large quantity of alkaloids.

Flavonoids are antioxidants with free radical scavenging ability and are therefore able to prevent oxidative damage in cells and have great anti-inflammatory activities [22], a basis for wound healing. Furthermore, flavonoids are also known to promote the wound healing process mainly due to their astringent and antimicrobial properties which are responsible for wound contraction and increased rate of epithelialization [23, 24]. Consequently, the wound healing ability of flavonoids has been observed to be even greater than that of silver sulfadiazine [25]. Flavonoids have also been observed to increase collagen synthesis, promote the cross-linking of collagen, shorten the inflammation period, and provide resistance against infections, important factors in enhancing the wound healing process [26]. These findings in part may be the reason behind the use of* A. africana* in the treatment of wounds, ulcers, and burns in traditional medicine.

Saponins' antioxidant and haemolytic properties make them one of the most important secondary metabolites in the treatment and management of a number of diseases, including wound healing [28, 29]. Indeed, saponins' ability to treat wounds and stop bleeding is due to the fact these phytochemicals precipitate and aggregate red blood cells [18]. Saponins are also known to enhance wound healing by causing wound contraction and bringing about high collagen deposition [29, 30]. In fact, saponins are also known to promote angiogenesis during skin wound repair [31]. Therefore, the high quantity of saponins in* A. africana* could explain why the plant has got such a potent ability to treat wounds in traditional medicine.

The presence of phenols in the plant leaf extract of* A. africana* is an indication that the extract may have antimicrobial properties [18] which greatly offers a basis for wound healing.

Tannins have been reported as having astringent activities which helps to quicken wound healing and treat inflammations [18]. Owing to its antibacterial activity and NIH3T3 cell proproliferative effect, tannins have been observed to promote wound contraction, improve healing rate, and promote healing of infectious wounds [32]. Specifically, tannins have been observed to reduce colonization of wounds by* S. aureus* resulting in a hasten wound healing [33]. Therefore, the presence of tannins may be one of the reasons why* A. africana* is renowned for wound healing in traditional medicine.

**Figure 4:**
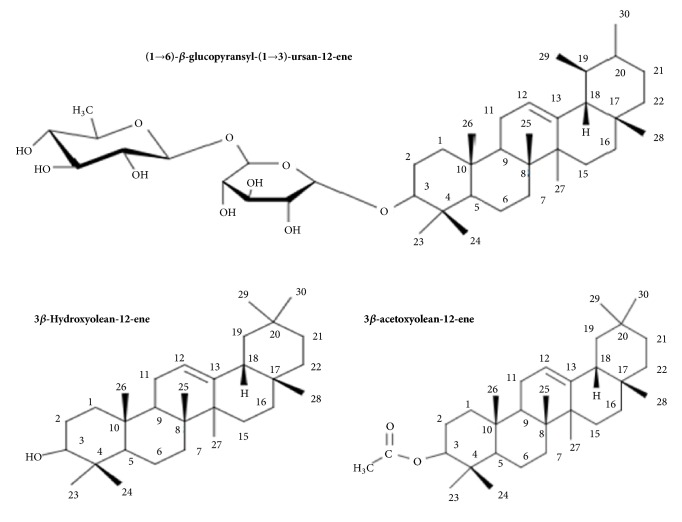
Isolated terpenoids from the leaves of* Aspilia africana *[27].

Terpenoids isolated from the leaves of* A. africana* include 3*β*-O-[*α*-rhamnopyranosyl-(1→6)-*β*-glucopyransyl-(1→3)-ursan-12-ene, 3*β*-Hydroxyolean-12-ene, and 3*β*-acetoxyolean-12-ene (Figure 4) [27]. Other terpenes present include *α*-pinene [34], carene, and phytol [19, 20] (Table 1).

Terpenoids are known to promote the wound healing process, mainly due to their astringent and antimicrobial properties, which seem to be responsible for wound contraction and an increased rate of epithelialization [35]. Carene (monoterpene) (Table 1) wound healing ability may be due to its antimicrobial activity in which it can inhibit the growth of* S. aureus* and* P. aeruginosa *in wounds [36–40]. Carene as an example of monoterpenes exhibited strong anti-inflammatory activity [41]. Therefore, the anti-inflammatory and antimicrobial activities of carene and other monoterpenes contained in* A. africana* somewhat validate the use of this plant in wound healing.

Alpha-pinene (Table 1) is an organic compound of the terpene class contained in* A. africana* [34, 42]. This vital compound was found to have potent anti-inflammatory activity [43]. The anti-inflammatory activity is due to its ability to suppress mitogen-activated protein kinases (MAPKs) and the nuclear factor-kappa B (NF-*κ*B) pathway which makes it a vital compound in the treatment of inflammatory diseases [44]. Beside its anti-inflammatory activity, singly or in synergy with other compounds, *α*-pinene has been observed to have interesting antimicrobial properties [46–48]. An* in vivo* study on* Pistacia atlantica* resin extract with 46.57%  *α*-pinene as the main content had a concentration-dependent effect on the healing of burn wounds after 14 days of treatment by increasing the concentration of basic fibroblast growth factor (BFGF), platelet derived growth factor, and improving angiogenesis [49]. Indeed, increased concentration of basic fibroblast growth factor is known to greatly enhance wound healing [49, 50]. Therefore, the antimicrobial, anti-inflammatory, and ability to increase BFGF level may explain why* A. africana *with *α*-pinene as one of the major compounds has been used in wound healing for generations.

Phytol (Table 1) is an acyclic diterpene alcohol with a percentage abundance of about 13% in the chloroform extract of* A. africana *[20]. This phytochemical has been shown to have wound healing activity. In an* in vivo* study, topical application of* Stachytarpheta jamaicensis* plant extract cream containing phytol on diabetic excision wound significantly improved (*P*<0.05) the percentage of wound contraction (88%) when compared to untreated diabetic rats in a period of 20 days [51]. It is important to note that wound healing can be greatly delayed due to infection by microorganisms [4].* Pseudomonas aeruginosa* is one of the most common bacteria isolated from chronic wounds and can express virulence factors on the surface proteins affecting wound healing [52]. Phytol is known to exert antibacterial property on* P. aeruginosa* via inducing oxidative stress [53]. Indeed, this compound is known to have high antimicrobial activity, high stability, and low toxicity [54]. In addition to the antimicrobial potential, phytol is also known to be one of the compounds with highly potent anti-inflammatory property [55, 56]. An* in vivo* study showed that phytol attenuated the inflammatory response by inhibiting neutrophil migration that is partly caused by reduction in interleukin-1*β* and tumor necrosis factor-*α* levels and oxidative stress [57]. The presence of phytol in* A. africana* therefore may explain why this plant has great antimicrobial and anti-inflammatory activities and hence its potent wound healing ability.

Caryophyllene (Table 1) is a natural bicyclic sesquiterpene that is a constituent of many essential oils belonging to a class of phytocannabinoids, one of the many compounds found in the extract of* A. africana *[19]. This compound has been shown to have potent antimicrobial property [58, 59]. Indeed, *β*-caryophyllene has demonstrated selective antibacterial activity against* S. aureus* (minimum inhibitory concentration (MIC) 3±1.0 *μ*M) and more pronounced antifungal activity [60]. Similarly, *β*-caryophyllene presented rapid bacterial killing for* S. aureus *(MIC <1.0 mg/Ml) in 4 h [61];* S. aureus* is one of the main microbial organisms that enhances wound sepsis [62]. *β*-caryophyllene has also been shown to exhibit great anti-inflammatory activity [63, 91, 92]. In a study by Dahham et al. [93], it was observed that *β*-caryophyllene elicited significant (*P*<0.01) reduction in paw volumes and low intensity of fluorescent signal in experimental animals when compared with negative control. Furthermore, the result indicated that the compound has a low toxicity, with high ability of skin penetration, greatly enhancing anti-inflammatory and analgesic activities making it useful for prevention and management of inflammation-related diseases, including wounds. Therefore, the antimicrobial and anti-inflammatory activities exhibited by *β*-caryophyllene contained in the extracts of* A. africana* could explain why this plant is so effective in wound healing.

Germacrene D (Table 1) is a volatile organic hydrocarbon compound belonging to the class sesquiterpenes contained in* A. africana* plant [27, 94, 95]. The compound possesses potent antimicrobial, anti-inflammatory, and antioxidant potentials activities [96–99]. Indeed germacrene D showed broad spectrum antibacterial activity against important human pathogenic Gram-positive and Gram-negative bacteria including* S. aureus *[100–102]. Therefore, the antimicrobial and anti-inflammatory activities exhibited by germacrene D contained in the extracts of* A. africana* could explain why this plant is so effective in wound treatment and management. However, more studies on isolated germacrene D needs to be conducted to validate further its wound healing potential.

Linolenic acid (Table 1) has been reported to have very strong antimicrobial activity against a number of microbes including those known to infect wounds and delay its healing such as* S. aureus* [103]. In addition, it is also an important anti-inflammatory agent [104]. Linolenic acid has been observed to down regulate inflammatory inducible nitric oxide synthase (iNOS), cyclooxygenase-2, and tumor necrosis factor-alpha gene expressions through the blocking of nuclear factor-kappaB and mitogen-activated protein kinases activation in lipopolysaccharide-stimulated murine macrophages cell line (RAW 264.7 cells), which may be the mechanistic basis for the anti-inflammatory effect of linolenic acid [105]. The presence of linolenic acid in* A. africana* therefore may explain why this plant has great antimicrobial and anti-inflammatory activities and hence its potent wound healing ability.

Through synergistic interactions of the different phytochemicals in* A. africana, *the plant has exhibited very strong antimicrobial, anti-inflammatory, and antioxidant activities which are vital components of the wound healing processes.

## 7. Conclusion

Throughout the world, wounds impose significant health burdens on millions of people. Consequently, all possible measures have to be taken to tackle it. Natural products have been used over the years for treatment and management of wounds.* A. africana* is one of the plants with immense attributes to enhance wound healing. The synergistic effects of the major phytochemicals in* A. africana* including alkaloids, saponins, tannins, flavonoids, *β*-caryophyllene, germacrene D, *α*-pinene, carene, phytol, and linolenic acid confer potent anti-inflammatory, antimicrobial, and antioxidant activities on the plant. This probably explains why this plant has such a potent wound healing ability. However, due to the reported adverse effects on the reproductive organs of the experimental animal models when administered orally, we recommend that future clinical studies focus on its topical application for wounds. Furthermore, although several studies have been carried out regarding chemical screening in* A. africana*, our review did not find any study on major nonvolatile chemical isolation and structure determination except for a limited study on terpenoids. Therefore, further studies on* A. africana* need to be done in this regard. Future studies also need to focus on the wound healing potential of the individual isolated compounds in* A. africana*. Furthermore, more preclinical and subsequently clinical studies need to be done to validate and understand the mechanism(s) of action of these phytochemicals in* A. africana* either in isolation or in combination for possible future wound healing drug development.

## Figures and Tables

**Table 1:** Constituent compounds in *A. africana* extract and associated activities that enhance wound healing.

**S/No**	**Class of compound**	**Phytochemical compounds**	**Compound structure**	**Activities that enhances wound healing**	**Reference**
**a**	Monoterpenes	carene	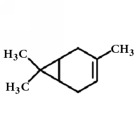	(i) Anti-inflammatory (ii) Antimicrobial	[19–26]

**b**	Phytocannabinoids	Caryophyllene	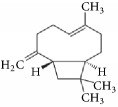	(i) Antimicrobial (ii) Anti-inflammatory	[19, 27–35]

**c**	Sesquiterpenes	Germacrene D	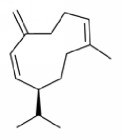	(i) Anti-inflammatory (ii) Anti-microbial and (iii) Anti-oxidant	[36–44]

**d**	Terpene	*α*-pinene	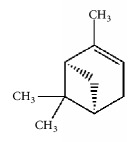	(i) Anti-microbial (ii) Anti-inflammatory (iii) Increases basic fibroblast growth factor (BFGF) (iv) Increases platelet derived growth factor	[45–53]

**e**	Acyclic diterpene alcohol	Phytol	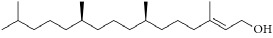	(i) Induces oxidative stress on microbial organisms (ii) Reduces interleukin-1*β* and tumor necrosis factor-*α* levels	[4, 20, 54–60]

**f**	Fatty *acid*	Linolenic acid	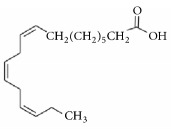	(i) Anti-microbial (ii) Down regulate inflammatory inducible nitric oxide synthase (iNOS).	[20, 61–63]
